# On the inscrutable role of Inscuteable: structural basis and functional implications for the competitive binding of NuMA and Inscuteable to LGN

**DOI:** 10.1098/rsob.120102

**Published:** 2012-08

**Authors:** Marina Mapelli, Cayetano Gonzalez

**Affiliations:** 1 Department of Experimental Oncology, European Institute of Oncology, Via Adamello 16, 20139 Milan, Italy; 2 Cell Division Group, IRB-Barcelona, PCB, c/Baldiri Reixac 10-12, Barcelona, Spain; 3 Institució Catalana de Recerca i Estudis Avançats (ICREA), Passeig Lluís Companys 23, Barcelona, Spain

**Keywords:** spindle orientation, asymmetric cell division, structural biology

## Abstract

Alignment of the mitotic spindle to the cellular polarity axis is a prerequisite for asymmetric cell divisions. The protein network coordinating the spindle position with cortical polarity includes the molecular machinery pulling on astral microtubules, which is assembled on conserved NuMA:LGN:Gαi complexes, the polarity proteins Par3:Par6:aPKC and an adaptor molecule known as Inscuteable (Insc). To date, all these components were assumed to enter a macromolecular complex localized at polarity sites in mitosis. However, recent structural studies revealed the Insc and NuMA are mutually exclusive interactors of LGN, implying that the molecular mechanism of spindle coupling to polarity is more sophisticated than has been believed to date.

## 2. Introduction

**Figure 1.**
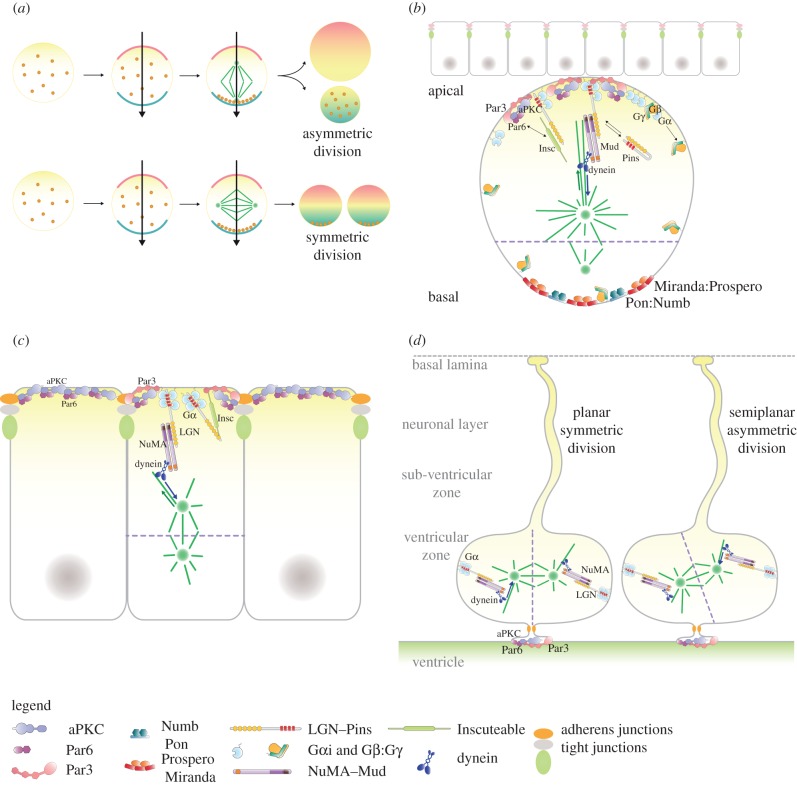
(*a*) Schematic definition of symmetric and asymmetric cell divisions. A prerequisite for asymmetric cell divisions is the establishment of a cellular polarity axis (black arrow), which entails the recruitment and maintenance at restricted cortical sites of defined sets of membrane-associated proteins (blue and pink crescent). Polarity establishment can be either a *self-autonomous* process occurring in early mitosis, as is the case for *Drosophila* neuroblasts and *Caenorhabditis elegans* zygotes, or it can be instructed from the tissue in which the cell is embedded. As mitosis proceeds, the proteins organized in distinct cortical domains (including the conserved Par3, Par6 and aPKC polarity proteins) coordinate the asymmetric distribution of fate determinants (i.e. components able to impart differential fate to daughter cells such as transcription factors and mRNAs—brown dots). In this configuration, if the mitotic spindle (in green) aligns parallel to the polarity axis, sibling cells will inherit a differential set of components (top panel). Furthermore, if one of the mother cell's cortical domains is in contact with a specific microenvironment (generally referred to as a *niche*), only one of the daughters will retain a similar contact. In addition, if the spindle is not central to the cell but displaced towards one side, the cytokinesis furrow will not ingress at the equatorial section, and upon cytokinesis daughters with different sizes will be generated (top panel). Conversely, if the spindle orients perpendicularly to the polarity axis, the division will be symmetrical, giving rise to two identical siblings (bottom panel). (*b*) Distribution of polarity proteins (Par3:Par6:aPKC), spindle orientation machinery (dynein-bound Mud:Pins:Gαi complexes) and of the bridging molecule dInsc in fruitfly neuroblasts at metaphase. Before division, neuroblasts delaminate from a neuroepithelium (top cell layer), to which they remain attached with a membrane region organizing the apical domain. Par proteins restrict the localization of fate determinants such as Prospero and Numb at the basal site. Despite the known force-generating complexes localizing apically, in neuroblasts the spindle is displaced towards the basal site in such a way that the cleavage plane (purple dotted line) parts the cytoplasm unequally. (*c*) During epidermal development, progenitors organized in a monolayered epithelium divide vertically to stratify the skin. mInsc mediates the recruitment of NuMA:LGN:Gαi at the apical site in order to properly orient the mitotic spindle. (*d*) Vertebrate neural stem cells, known as radial glial cells, undergo planar symmetric divisions (left) and semiplanar asymmetric divisions (right), with a proportion that is finely regulated throughout neurogenesis. Planar divisions occur with the spindle axis parallel to the ventricular surface, and the cleavage plane bisecting both the apical end-foot and the basal process. A minor tilt in the spindle axis is sufficient for the asymmetric segregation of the basal process and the apical end-foot between daughters. In radial glial cells, NuMA:LGN:Gαi complexes localize in an equatorial belt, away from Par proteins, which are found in the apical end-foot. Although mInsc regulates the balance between planar and semiplanar divisions of radial glia, its compartmentalization in mitosis is not yet clear.

The asymmetric outcome of a cell division relies on the tight coordination between cortical polarity and the orientation of the mitotic spindle. During asymmetric mitoses, membrane-associated proteins distribute in discrete cortical domains, establishing a cellular polarity axis. In this configuration, the position of the mitotic spindle, and hence of the cytokinesis plane, determines whether the two daughter cells will (i) inherit the same set of cellular components, (ii) retain analogous contacts to the external tissue and (iii) have the same size (figure 1*a*).

Our understanding of the basic mechanisms underlying spindle coupling to cortical polarity in asymmetric divisions greatly benefited from genetic studies conducted in the late 1980s in model systems such as *Drosophila* neuroblasts (the stem cells of the central nervous system in flies) and *Caenorhabditis elegans* zygotes [1]. In particular, fly neuroblasts undergo self-renewing asymmetric divisions, generating one neuroblast and one ganglion mother cell destined for differentiation. After delamination from the neuroepithelium, mitotic neuroblasts organize an apical cortical domain where the polarity proteins Bazooka(Par3):Par6:aPKC are clustered, and a basal cortical crescent where the fate determinants Numb and Prospero are confined (figure 1*b*). Par complexes recruit at the apical site macromolecular machines known as *force generators*, able to capture astral microtubules emanating from the spindle poles and to establish pulling forces. Genetic screens combined with imaging studies in developing fly embryos and larval brains revealed that force generators are assembled on Mud:Pins:Gαi complexes brought at sites of polarization by an adaptor named Inscuteable (hereafter referred to as dInsc, where ‘d’ stands for *Drosophila*) that directly interacts with Bazooka (the fly Par3) [2–5]. The traction force exerted on microtubules is generated by the minus-end-directed movement of Dynein/Dynactin that directly interacts with Mud [6]. As a result of the directional pulling forces exerted by force generators, the spindle aligns parallel to the polarity axis, slightly displaced towards the basal site. Upon cytokinesis, the fate determinants partition only to the smaller ganglion mother cell, which is positioned away from the neuroepithelium. Genetic studies revealed that the size difference between the neuroblast and the ganglion mother cell, and the unequal segregation of Prospero, are fundamental events in setting the correct lineage length and progeny fate [7].

Insights into the mechanistic basis of spindle coupling to cortical polarity in vertebrates have lagged behind those in flies and worms, mainly due to technical difficulties in isolating and studying stem cells. Yet recent evidence supports the notion that the spindle orientation machinery active in neuroblasts is conserved in mouse skin and neural progenitors, and is responsible for vertical divisions in lung distal epithelium [8]. At about day E12.5 of embryonic mouse development, progenitors of the epidermis start dividing vertically to promote skin stratification. Such vertical asymmetric divisions are promoted by the apical recruitment of NuMA:LGN:Gαi complexes (the vertebrate counterpart of Mud:Pins:Gαi) by Par6:Par3:aPKC via mInsc (where ‘mInsc’ indicates the *m*ammalian orthologue of dInsc), which results in apico-basal spindle alignment and generates one daughter cell placed above the epidermal layer (figure 1*c*) [9,10]. The situation is more sophisticated in neural stem cells, called radial glial cells in vertebrates. Radial glial cells are attached to the ventricular zone through a tiny apical end-foot, accounting for only about 2 to 3 per cent to the total cellular membrane, where Par proteins localize [11,12]. Seminal studies from the Huttner and Ffrench-Constant laboratories [13,14] revealed that in early embryogenesis apical neural progenitors mostly divide planarly, with the spindle parallel to the ventricular surface (figure 1*d*, left panel). Between E13 and E18, when mouse neurogenesis peaks, radial glial cells divide semi-planarly with a minor tilt in the spindle axis that is sufficient to determine the unequal inheritance of the apical foot domain and the basal process (figure 1*d*, right panel). Imaging analysis in the developing chicken neocortex revealed that NuMA:LGN:Gαi are distributed in an equatorial belt above the spindle poles, and do not seem to co-localize with Par proteins [15]. No clear evidence is available for the precise cortical localization of mInsc in radial glial cells, although it has been shown that its overexpression in mice at day E8.5 increases tilted neurogenic divisions, whereas its ablation causes radial glial expansion [16]. The prominent role of planar divisions in maintaining the population of apical neural progenitors in the developing central nervous system is also supported by knock-down experiments of LGN in chicken and mouse neuroepithelial cells, which result in randomization of the spindle axis and premature exit from the neuroepithelium, but do not compromise the replicative potential [17,18].

What do we know about the molecular mechanisms accounting for spindle coupling to cortical polarity? As briefly sketched earlier, for years, the simple model that was pushed forward to explain spindle orientation was that Par proteins, mInsc, NuMA, LGN and Gαi would be part of the same macromolecular complex, apically localized in polarized asymmetric divisions, and capable of generating pulling forces on astral microtubules via the direct interaction between NuMA and the microtubule motor Dynein/Dynactin [19]. This model stems from imaging analyses showing that this set of proteins localizes in a crescent above the apical spindle pole, and was further corroborated by coimmunoprecipitation experiments in which LGN:Gαi were found in association with Par3:mInsc [20] and NuMA [21].

Recent biochemical and structural data revealed that mInsc and NuMA are in fact competitive interactors of LGN [22,23]. This evidence challenges the notion that they belong to the same complex, and imposes its revision on the basis of the newly acquired knowledge. Here, we review the organizational and functional principles of the interactions of mInsc and NuMA with LGN, and explain the molecular basis for their competition, emphasizing similarities and differences between *Drosophila* and vertebrate proteins. We also advance new hypotheses for the working principles of these proteins truly reflecting their biochemical properties.

## 3. Conserved architecture of LGN:Insc and Pins:Insc complexes

LGN and Pins share a homologous domain structure comprising eight tetratricopeptide repeats (TPRs) at the N-terminus and multiple GoLoco motifs at the C-terminus joined by a linker region of about 100 residues. TPRs consist of a couple of antiparallel α-helices, and are usually arranged in contiguous arrays forming superhelical scaffolds that mediate protein–protein interactions. Indeed, LGN^TPR^ has been shown to be responsible for the interaction with NuMA [21,24] and Lgl2 [25], and Pins^TPR^ associates with Mud [26–29], dInsc [4] and Canoe [30]. Recently, the topologies of binding of LGN^TPR^/Pins^TPR^ to mInsc/dInsc [22,31] and NuMA [23] have been unveiled.

**Figure 2.**
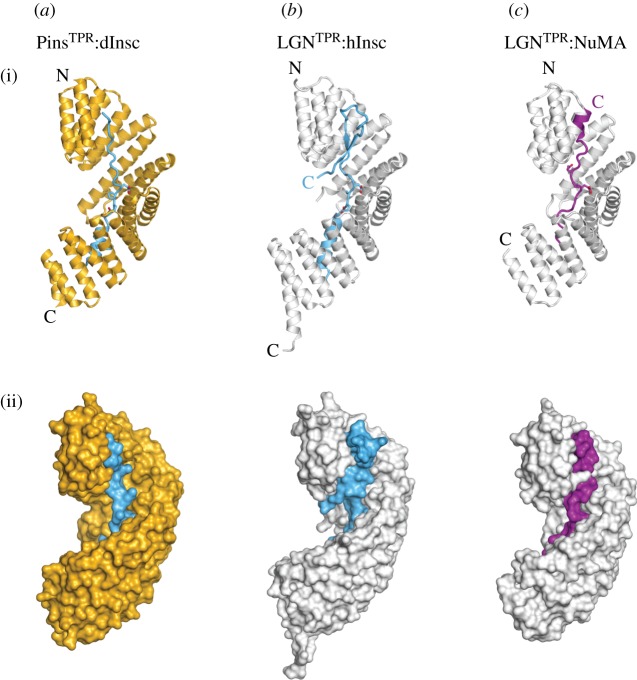
Architecture of Pins^TPR^/LGN^TPR^ in complex with Insc and NuMA. (*a*) (i) Cartoon and (ii) surface representation of *Drosophila* Pins^TPR^:dInsc^PEPT^ (PDB ID 4A1S). Pins is shown in yellow and dInsc in blue. (*b*) Structure of human LGN^TPR^:hInsc^PEPT^ (PDB ID 3SF4) displayed with the same orientation as in (*a*), with LGN coloured grey. The longer hInsc fragment forms a β-hairpin lining on the N-terminal TPRs of LGN. (*c*) A structure of mouse LGN^TPR^:NuMA^PEPT^ (PDB ID 3RO2), showing that hInsc and NuMA (purple) occupy the same surface in the inner groove of the TPR domain. The C-terminal portion of NuMA^PEPT^ adopts a helical conformation.

**Figure 3.**
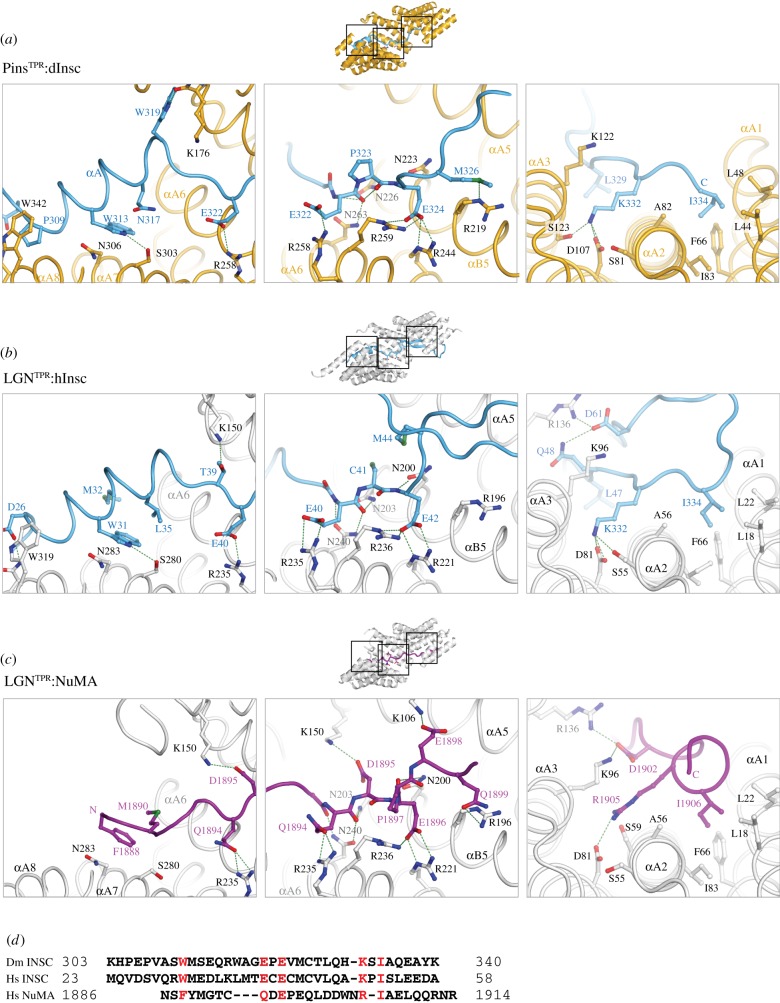
A structural comparison of the interaction surface of dInsc/hInsc and NuMA with Pins^TPR^/LGN^TPR^. (*a*) Enlarged views of the modular interface between dInsc^PEPT^ and Pins^TPR^. Conserved residues contributing to the dimer interface are shown in balls-and-sticks, including the EPE^Insc^-motif in the central portion of the peptide. (*b*) Analogous close-ups of human hInsc^PEPT^:LGN^TPR^, in which the additional C-terminal β-strand is visible. (*c*) Details of the NuMA^PEPT^-binding interface in the same LGN^TPR^ regions displayed in (*a*,*b*). (*d*) A structure-based sequence alignment of Insc^PEPT^ and NuMA^PEPT^ recapitulating the high-affinity interaction with LGN/Pins. Residues engaged in chemically equivalent interactions with the TPR scaffold are coloured in red.

The direct interaction of Pins/LGN with dInsc/mInsc is required for apico-basal spindle alignment in neuroblasts [2] and in mouse skin progenitors [9]. Deletion analysis revealed that the portion of fruitfly dInsc encompassing residues 252–615, termed asymmetric-domain or dInsc^ASYM^, is sufficient to recapitulate dInsc functions during neuroblast asymmetric divisions [4,32]. The poor sequence similarity among Insc homologues makes it difficult to identify *in silico* a corresponding portion on the vertebrate proteins. Nonetheless, comparison of the crystallographic structures of *Drosophila* Pins^TPR^:dInsc [22] with human and mouse LGN^TPR^:mInsc [23,31] revealed that the interface between the 40-residue peptide of Insc (Insc^PEPT^ hereafter) containing the high-affinity LGN-binding site and LGN^TPR^ is conserved throughout species (figure 2*a*,*b*). For clarity, we will use hInsc when referring to the *h*uman protein. The crystallographic structures revealed that Insc^PEPT^ adopts an extended conformation lining within the inner groove of the superhelical TPR scaffold of LGN/Pins with an opposite orientation. This arrangement defines a modular interface. The first 12 residues of Insc^PEPT^ fold as an α-helix that packs perpendicular to the TPRs and contribute to the binding strength primarily via hydrophobic interaction mediated by Trp313^dInsc^/Trp31^hInsc^ (figure 3*a*,*b*, left panels). The central portion of Insc^PEPT^ is anchored on Pins^TPR^/LGN^TPR^ by bidentate hydrogen bonds between Asn residues of the Leu–Gly–Asn (LGN) triplets of TPR4–5 and main chain carbonyls and nitrogen of Insc. In this region, the interaction is further strengthened by polar interactions between the negatively charged Glu-X-Glu motif of Insc^PEPT^ and invariant Arg side chains of Pins/LGN (figure 3*a*,*b*, central panels). The C-terminal portion of Insc^PEPT^ inserts Lys332^dInsc^/Lys50^hInsc^ and Ile334^dInsc^/Ile52^hInsc^ into two adjacent pockets formed by TPR1–2–3 (figure 3*a*,*b*, right panels). Although these lysine and isoleucine residues are fully conserved, their substitution does not affect the binding affinity. Interestingly, the structure of human LGN^TPR^:hInsc^PEPT^, which has been determined with a longer Insc fragment than the fly one, shows that the hInsc chain folds back into a β-hairpin (figure 2*b*, top panel), though this C-terminal extension does not seem to make specific contacts with LGN^TPR^. This C-terminal stretch is poorly conserved in *Drosophila* dInsc and was not protected by trypsin cleavage of Pins^TPR^:dInsc^ASYM^ [22], raising the question as to whether it adopts the same fold in the fly complex. In all species, the extended interaction surface accounts for the nanomolar binding affinity.

## 4. Ubiquitous functions of NuMa:LGN:Gαi in mitotic spindle orientation

In all mitoses, the placement of the mitotic spindle relies on the interaction between NuMA and cortically localized LGN:Gαi complexes [33]. In Hela cells, NuMA:LGN:Gαi are distributed above both poles to stabilize the spindle and assist sister chromatid separation in anaphase. Myristoylated Gαi subunits recruit the complex at the cortex [21], while its restricted localization above the poles relies on a Ran^GTP^-dependent gradient precluding membrane association in the proximity of the metaphase plate [34]. In this system as well as in skin progenitors, Abl-1 kinase orchestrates the coordination of LGN crescents with the spindle axis by phosphorylating NuMA on Tyr1774, with molecular details that are still unclear [35]. In oriented symmetrical divisions of epithelial cells, NuMA:LGN:Gαi complexes are restricted baso-laterally in order to keep the spindle horizontal, and do not localize with Par proteins. The mechanism that has been put forward to account for the apical exclusion of NuMA:LGN:Gαi in this system is the phosphorylation of Ser401^LGN^ by apical aPKC that primes the association of LGN with 14-3-3, thus precluding the binding to cortical Gαi [36].

In polarized asymmetric mitosis, the NuMA:LGN:Gαi complex is thought to be recruited apically with Par3/Par6/aPKC via mInsc to unequally partition fate determinants. As previously outlined, this mechanism has been pushed forward to explain the spindle alignment to cortical polarity in fly neuroblasts and mouse skin progenitors, whereas radial glial cells seem to constitute an exception to this simplistic view. In our opinion, this apparent discrepancy can be reconciled, envisioning that a few mInsc-coordinated NuMA:LGN:Gαi complexes localized with Par proteins at the apical end-foot, but not detectable by immunostaining, might be sufficient to tilt the spindle axis even if the bulk of NuMA:LGN:Gαi is on an equatorial belt [15] (figure 1*d*). Interestingly, in asymmetrically dividing *Drosophila* neuroblasts, the phosphorylation of Ser436^Pins^ (corresponding to Ser401^LGN^) by Aurora-A has been reported to trigger a secondary microtubule-dependent spindle orientation pathway sustained by the phospho-specific association of Pins with membrane-associated Dlg [23,37,38], an association that can also occur when Pins is in complex with dInsc [39]. There is no clear evidence as to whether the pathway is conserved in vertebrates. In fruitflies’ neuroblasts, an additional player of the spindle orientation network is the actin-binding protein Canoe [40], which directly binds Pins, and has been implicated in Mud recruitment at the cortex in a Ran^GTP^-dependent manner [30]. *In vitro* Canoe interacts directly with Ran^GTP^; however, how this association may favour the localization of Mud is still unclear. It will be extremely interesting to test whether the orthologue Afadin plays an analogous role in vertebrates' asymmetric divisions, as this interaction represents the first direct link between the acto-myosin cortex and the spindle-tethering machinery.

## 5. Structural organization of the NuMa:LGN assembly

It has long been known from biochemical studies that a 20 kDa C-terminal fragment of NuMA contains the LGN^TPR^-binding site [24], which partly overlaps with the microtubule-binding site [41], thus implying that the microtubule-organizing activity of NuMA and its function in spindle orientation are mutually exclusive. Recent structural evidence revealed that NuMA contacts the internal concave surface of LGN^TPR^ with a short amino acidic stretch spanning residues 1886–1914 of the human protein (which we will refer to as NuMA^PEPT^), roughly occupying the same surface recognized by Insc^PEPT^ (figure 2*c*) [23]. At the molecular level, NuMA^PEPT^ runs parallel to the TPR superhelical axis, forming with its C-terminus a three-helix bundle with αA1–αA2 of LGN (figure 3*c*). At first glance, the only conserved motif between Insc^PEPT^ and NuMA^PEPT^ sequences is the central Glu-X-Glu motif. However, a careful inspection of the dimeric interface shows that several residues of the two peptides with analogous interacting potential are engaged in the same type of chemical bonds, and that their contributions sum up to create a substantially identical interaction network with LGN^TPR^ (figure 3*c*). In particular, the Phe1888^NuMA^ ring is positioned as Trp31^hInsc^/Trp313^dInsc^, whereas Arg1905^NuMA^ and Ile1906^NuMA^ insert into negatively charged and hydrophobic cavities of LGN^TPR^ in much the same way as Lys50^hInsc^/Lys332^dInsc^ and Ile52^hInsc^/Ile334^dInsc^. Notably, the Glu–Pro–Glu motif of NuMA^PEPT^ is two residues out of register when compared with the Glu-X-Glu^Insc^, which structurally aligns with the upstream Gln–Asp–Glu^NuMA^ triplet (figure 3*c*,*d*). As Insc^PEPT^ and NuMA^PEPT^ occupy the same position on LGN^TPR^, we expect that designing LGN^TPR^ mutants selectively impairing the binding either to mInsc or to NuMA will be a challenging exercise. It is worth pointing out that the common molecular signature described for the recognition of mInsc/dInsc and NuMA by LGN^TPR^/Pins^TPR^ has not allowed the identification of a corresponding Pins-binding peptide on Mud. Consistent with the structural analysis, NuMA also binds LGN^TPR^.

## 6. NuMa and mInsc bind competitively to LGN^TPR^

The evidence that mInsc and NuMA share the same binding surface on LGN^TPR^ depicts them as competitive interactors. Indeed, in several experimental settings, it has been proved that *in vitro* and in living cells the two proteins do not enter the same complex [22,23,39]. Measurements with human proteins suggest that Insc^PEPT^ associates with LGN^TPR^ with a fivefold to tenfold higher affinity than NuMA^PEPT^ [22,31], whereas mouse proteins display similar binding strengths [23]. Although no quantification is available, full-length LGN:Gαi also displays a preferential association with full-length mInsc than with NuMA [22]. The relation seems to be opposite for *Drosophila* proteins as Pins binds Mud better [39]. How the relative affinities impinge on the crosstalk between the mInsc-bound and the NuMA-bound LGN:Gαi pools in living cells remains to be determined.

## 7. Opening of the LGN conformational switch

Besides competing with one another for the binding to LGN^TPR^, both NuMA and mInsc are capable of displacing the C-terminal portion of LGN, which has been shown to interact with the TPR domain in unliganded LGN [21,22,29]. The precise fragment of LGN C-terminus required for this intra-molecular interaction has not been mapped; what it is known is that the GoLoco23 of Pins [29] and GoLoco34 of LGN (M. Mapelli 2011, unpublished data) form a complex with LGN^TPR^. On the basis of recent biochemical and structural data, we suspect LGN^TPR^ has an inherent conformational flexibility that needs to be stabilized by association with an extended ligand, and therefore we expect the LGN C-terminus to bind LGN^TPR^ with modalities similar to the those described for mInsc and NuMA. The affinity between separately purified LGN^TPR^ and GoLocos is in the micromolar range [29] (M. Mapelli 2011, unpublished data), suggesting that substoichiometric concentrations of NuMA and mInsc might be sufficient for the LGN switch conversion. Intriguingly, *in vivo* the activation of full-length LGN and Pins requires the synergistic binding of NuMA/Mud and several Gαi subunits [42,43], implying an active role of the linker region in maintaining LGN in the closed state. It is not known whether mInsc can trigger the LGN conformational transition in the absence of Gαi. As the release of the intra-molecular interaction holding LGN in an inhibited closed conformation is a prerequisite for force generators' assembly, we believe that elucidating the structural role of the linker region in closed LGN will be instrumental in figuring out the LGN activation mechanism.

## 8. A revised model for force generators' assembly in asymmetric cell divisions

**Figure 4.**
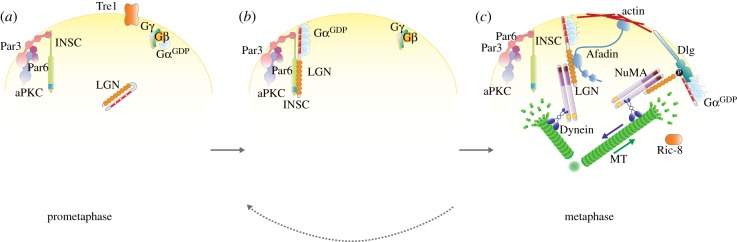
A sequential model for force generators' recruitment and maintenance at polarized cortical sites. (*a*) In early mitosis, Par3:Par6:aPKC localize in an apical cortical domain together with mInsc that binds directly to Par3. Cytosolic LGN is in the inactive closed conformation, and hetero-trimeric G-protein complexes are uniformly distributed all around the plasma membrane. (*b*) As mitosis proceeds, LGN is recruited to Par proteins by the high-affinity interaction with mInsc, which triggers the switch-like conformational transition enabling the binding of four Gαi^GDP^ subunits previously disengaged from Gβγ. Gα dissociation from Gβγ may be assisted by a specific G-protein coupled receptor such as the *Drosophila* Tre1. (*c*) At metaphase, most of the membrane-associated LGN:Gαi^GDP^ is released from mInsc and enters a complex with dimeric NuMA, which in turn interacts with the minus-end-directed motor Dynein to pull on astral microtubules (MTs). At this stage, cortical anchoring of NuMA:LGN:Gαi^GDP^ complexes can be strengthened by interactions between Ser406-phosphorylated LGN and Dlg, or between LGN and actin-bound Afadin. On the basis of the sequential model, NuMA:LGN:Gαi^GDP^ assemble near Par complexes, but do not retain any positional information. To prevent mis-directional MT pulling caused by membrane-diffusion of NuMA:LGN:Gαi^GDP^, it is possible that these complexes are short-lived and disassemble under the action of the Gαi GEF Ric-8, and that new cycles of LGN recruitment by mInsc start until the spindle is properly aligned (dotted arrow).

The competitive association of NuMA and mInsc with LGN contrasts with the notion that mInsc is the molecular bridge recruiting NuMA:LGN:Gαi to polarity sites during asymmetric mitoses, and imposes a major revision of the current view for force generators' localization and maintenance at the cortex. The more simplistic model that has been advanced [22,23] envisions the sequential binding of LGN first to Par3-bound mInsc and subsequently to NuMA (figure 4). In this view, in the early phases of mitosis, the higher affinity interaction with mInsc will be dominant in instructing the cortical distribution of LGN, and concomitantly will catalyse the conformational rearrangement required for the binding to several Gαi subunits. If so, the functional role of mInsc would be to cluster and activate LGN at the membrane, a known mechanism able to trigger localized signalling pathways [44]. How the transfer of LGN:Gαi complexes from mInsc to NuMA during mitosis might be achieved remains totally unclear. Biochemical measurements indicate that there is about a fivefold difference in the binding strength of mInsc and NuMA to LGN, which we suggest could be overcome by addition of a few phospho-groups either to NuMA or LGN. None of the mitotic phosphorylations of NuMA recently reported affects the interaction with LGN [34,35,45,46], though a clear function has been assigned only to a few of them. An inherent question of the sequential-binding model is how the NuMA:LGN:Gαi complexes that are assembled in the proximity of Par proteins are retained long enough at the correct sites to pull on astral microtubules with the proper direction. As mentioned, in fly neuroblasts, cortical Dlg binds to the phosphorylated linker region of Pins, providing an anchoring mechanism independent of Par proteins [38]. We believe that newly identified Pins/LGN interactors (such as the earlier-mentioned Canoe, as well as yet undiscovered effectors) may serve a similar purpose. Interestingly, ablation of α-catenin and β-integrin in skin progenitors causes misoriented divisions, implying that other signalling pathways not directly regulated by Par3:aPKC contribute to spindle positioning in this system. Alternatively, NuMA:LGN:Gαi can be transient complexes, briefly pulling in the direction of Par proteins where they are assembled, and then dissociating before diffusing away by spontaneous drift of the Gαi myristoyl groups inserted in the plasma membrane. It is known that LGN binds exclusively to GDP-loaded Gαi [47], and that the conserved GEF Ric-8 catalyses the GDP exchange of Gαi, which is essential to the spindle orientation process [48–51]. In the context of the sequential-binding model, the function of Ric-8 could be to assist the disruption of the NuMA:LGN:Gαi^GDP^ to start a new mInsc-binding cycle, a molecular mechanism already observed *in vitro* [52]. An additional role for the non-canonical G-protein signalling pathway underlying spindle orientation has been recently described in *Drosophila* neuroblasts, where Pins has been reported to bind not only Gαi^GDP^ but also Goα^GDP^ [53]. In this system, the regulation of the Pins:Goα^GDP^ association by the G-protein coupled receptor Tre1 constitutes an extrinsic cue coordinating cortical polarity with tissue architecture as it instructs the organization of the apical domain at the site in contact with the neuroepithelium.

The validation of this sequential/transient-binding model in living cells would greatly benefit from the availability of LGN mutants selectively impaired for mInsc or NuMA binding. The model predicts that LGN mutants unable to bind mInsc would phenocopy the spindle misorientation observed in dInsc/mInsc-deficient neuroblasts or radial glia cells, whereas mutants deficient in NuMA binding would be recruited to Par proteins but fail to pull on astral microtubules. Furthermore, these selective mutations could be introduced into FRET sensors (such as the already-used YFP-LGN/Pins-CFP sensors [21,29]) to check whether the opening of the switch occurs upon Insc or NuMA binding. Given the major overlap between mInsc and NuMA binding sites, we anticipate that generating such LGN mutants will be a rather daunting task.

## 9. Conclusions

In the past few years, the prominent role of the spindle alignment in setting the balance between symmetric and asymmetric divisions of stem cells has fuelled intense research activities aimed at understanding the molecular mechanisms underpinning the process in several cellular systems. It is now clear that in fly neuroblasts, intrinsic and extrinsic cues synergize in positioning an asymmetric spindle, and this is a prerequisite for the correct fate and placement of daughter cells [7,53]. Attempts have been made to recapitulate in vertebrates what is known from genetic and imaging studies in fly neuroblasts, and this effort led to the conclusion that randomizing the progenitors' spindle orientation impacts on neurogenetic programmes also in vertebrates [16–18]. Recent biochemical and structural evidence indicated an inconsistency in the established molecular model for spindle orientation, requiring more analyses to clarify the mechanistic interplay between mInsc and NuMA. Meanwhile, the number of interactors of the NuMA:LGN:Gαi complex and the way they regulate the spindle orientation in different cellular systems starts to become clear. This sets the stage for further investigations *in vivo* and *in vitro* aimed at delivering a coherent picture of how they function.

## References

[1] Siller KH Doe CQ 2009 Spindle orientation during asymmetric cell division. Nat. Cell Biol. 11, 365–374 10.1038/ncb0409-365 (doi:10.1038/ncb0409-365) 19337318

[2] Kraut R Chia W Jan LY Jan YN Knoblich JA 1996 Role of Inscuteable in orienting asymmetric cell divisions in *Drosophila*. Nature 383, 50–55 10.1038/383050a0 (doi:10.1038/383050a0) 8779714

[3] Kraut R Campos-Ortega JA 1996 Inscuteable, a neural precursor gene of *Drosophila*, encodes a candidate for a cytoskeleton adaptor protein. Dev. Biol. 174, 65–81 10.1006/dbio.1996.0052 (doi:10.1006/dbio.1996.0052) 8626022

[4] Yu F Morin X Cai Y Yang X Chia W 2000 Analysis of partner of Inscuteable, a novel player of *Drosophila* asymmetric divisions, reveals two distinct steps in Inscuteable apical localization. Cell 100, 399–409 10.1016/S0092-8674(00)80676-5 (doi:10.1016/S0092-8674(00)80676-5) 10693757

[5] Schaefer M Shevchenko A Shevchenko A Knoblich JA 2000 A protein complex containing Inscuteable and the Gα-binding protein Pins orients asymmetric cell divisions in *Drosophila*. Curr. Biol. 10, 353–362 10.1016/S0960-9822(00)00401-2 (doi:10.1016/S0960-9822(00)00401-2) 10753746

[6] Wang C 2011 An ana2/ctp/mud complex regulates spindle orientation in *Drosophila* neuroblasts. Dev. Cell 21, 520–533 10.1016/j.devcel.2011.08.002 (doi:10.1016/j.devcel.2011.08.002) 21920316

[7] Kitajima A Fuse N Isshiki T Matsuzaki F 2010 Progenitor properties of symmetrically dividing *Drosophila* neuroblasts during embryonic and larval development. Dev. Biol. 347, 9–23 10.1016/j.ydbio.2010.06.029 (doi:10.1016/j.ydbio.2010.06.029) 20599889

[8] El-Hashash AH Warburton D 2011 Cell polarity and spindle orientation in the distal epithelium of embryonic lung. Dev. Dyn. 240, 441–445 10.1002/dvdy.22551 (doi:10.1002/dvdy.22551) 21246661 PMC3023987

[9] Williams SE Beronja S Pasolli HA Fuchs E 2011 Asymmetric cell divisions promote Notch-dependent epidermal differentiation. Nature 470, 353–358 10.1038/nature09793 (doi:10.1038/nature09793) 21331036 PMC3077085

[10] Lechler T Fuchs E 2005 Asymmetric cell divisions promote stratification and differentiation of mammalian skin. Nature 437, 275–280 10.1038/nature03922 (doi:10.1038/nature03922) 16094321 PMC1399371

[11] Farkas LM Huttner WB 2008 The cell biology of neural stem and progenitor cells and its significance for their proliferation versus differentiation during mammalian brain development. Curr. Opin. Cell Biol. 20, 707–715 10.1016/j.ceb.2008.09.008 (doi:10.1016/j.ceb.2008.09.008) 18930817

[12] Gotz M Huttner WB 2005 The cell biology of neurogenesis. Nature reviews. Mol. Cell Biol. 6, 777–788 10.1038/nrm1739 16314867

[13] Kosodo Y Roper K Haubensak W Marzesco AM Corbeil D Huttner WB 2004 Asymmetric distribution of the apical plasma membrane during neurogenic divisions of mammalian neuroepithelial cells. EMBO J. 23, 2314–2324 10.1038/sj.emboj.7600223 (doi:10.1038/sj.emboj.7600223) 15141162 PMC419905

[14] Marthiens V Ffrench-Constant C 2009 Adherens junction domains are split by asymmetric division of embryonic neural stem cells. EMBO Rep. 10, 515–520 10.1038/embor.2009.36 (doi:10.1038/embor.2009.36) 19373255 PMC2680875

[15] Peyre E Jaouen F Saadaoui M Haren L Merdes A Durbec P Morin X 2011 A lateral belt of cortical LGN and NuMA guides mitotic spindle movements and planar division in neuroepithelial cells. J. Cell Biol. 193, 141–154 10.1083/jcb.201101039 (doi:10.1083/jcb.201101039) 21444683 PMC3082188

[16] Postiglione MP Juschke C Xie Y Haas GA Charalambous C Knoblich JA 2011 Mouse Inscuteable induces apical–basal spindle orientation to facilitate intermediate progenitor generation in the developing neocortex. Neuron 72, 269–284 10.1016/j.neuron.2011.09.022 (doi:10.1016/j.neuron.2011.09.022) 22017987 PMC3199734

[17] Morin X Jaouen F Durbec P 2007 Control of planar divisions by the G-protein regulator LGN maintains progenitors in the chick neuroepithelium. Nat. Neurosci. 10, 1440–1448 10.1038/nn1984 (doi:10.1038/nn1984) 17934458

[18] Konno D Shioi G Shitamukai A Mori A Kiyonari H Miyata T Matsuzaki F 2008 Neuroepithelial progenitors undergo LGN-dependent planar divisions to maintain self-renewability during mammalian neurogenesis. Nat. Cell Biol. 10, 93–101 10.1038/ncb1673 (doi:10.1038/ncb1673) 18084280

[19] Merdes A Heald R Samejima K Earnshaw WC Cleveland DW 2000 Formation of spindle poles by dynein/dynactin-dependent transport of NuMA. J. Cell Biol. 149, 851–862 10.1083/jcb.149.4.851 (doi:10.1083/jcb.149.4.851) 10811826 PMC2174573

[20] Zigman M 2005 Mammalian Inscuteable regulates spindle orientation and cell fate in the developing retina. Neuron 48, 539–545 10.1016/j.neuron.2005.09.030 (doi:10.1016/j.neuron.2005.09.030) 16301171

[21] Du Q Macara IG 2004 Mammalian Pins is a conformational switch that links NuMA to heterotrimeric G proteins. Cell 119, 503–516 10.1016/j.cell.2004.10.028 (doi:10.1016/j.cell.2004.10.028) 15537540

[22] Culurgioni S Alfieri A Pendolino V Laddomada F Mapelli M 2011 Inscuteable and NuMA proteins bind competitively to Leu-Gly-Asn repeat-enriched protein (LGN) during asymmetric cell divisions. Proc. Natl Acad. Sci. USA 108, 20 998–21 003 10.1073/pnas.1113077108 (doi:10.1073/pnas.1113077108) PMC3248549 22171003

[23] Zhu J 2011 LGN/mInsc and LGN/NuMA complex structures suggest distinct functions in asymmetric cell division for the Par3/mInsc/LGN and Gαi/LGN/NuMA pathways. Mol. Cell 43, 418–431 10.1016/j.molcel.2011.07.011 (doi:10.1016/j.molcel.2011.07.011) 21816348 PMC3158460

[24] Du Q Stukenberg PT Macara IG 2001 A mammalian partner of Inscuteable binds NuMA and regulates mitotic spindle organization. Nat. Cell Biol. 3, 1069–1075 10.1038/ncb1201-1069 (doi:10.1038/ncb1201-1069) 11781568

[25] Yasumi M Sakisaka T Hoshino T Kimura T Sakamoto Y Yamanaka T Ohno S Takai Y 2005 Direct binding of Lgl2 to LGN during mitosis and its requirement for normal cell division. J. Biol. Chem. 280, 6761–6765 10.1074/jbc.C400440200 (doi:10.1074/jbc.C400440200) 15632202

[26] Siller KH Cabernard C Doe CQ 2006 The NuMA-related Mud protein binds Pins and regulates spindle orientation in *Drosophila* neuroblasts. Nat. Cell Biol. 8, 594–600 10.1038/ncb1412 (doi:10.1038/ncb1412) 16648843

[27] Izumi Y Ohta N Hisata K Raabe T Matsuzaki F 2006 *Drosophila* pins-binding protein Mud regulates spindle-polarity coupling and centrosome organization. Nat. Cell Biol. 8, 586–593 10.1038/ncb1409 (doi:10.1038/ncb1409) 16648846

[28] Bowman SK Neumuller RA Novatchkova M Du Q Knoblich JA 2006 The *Drosophila* NuMA homolog Mud regulates spindle orientation in asymmetric cell division. Dev. Cell 10, 731–742 10.1016/j.devcel.2006.05.005 (doi:10.1016/j.devcel.2006.05.005) 16740476

[29] Nipper RW Siller KH Smith NR Doe CQ Prehoda KE 2007 Gαi generates multiple Pins activation states to link cortical polarity and spindle orientation in *Drosophila* neuroblasts. Proc. Natl Acad. Sci. USA 104, 14 306–14 311 10.1073/pnas.070812104 (doi:10.1073/pnas.070812104) PMC1964812 17726110

[30] Wee B Johnston CA Prehoda KE Doe CQ 2011 Canoe binds RanGTP to promote Pins(TPR)/Mud-mediated spindle orientation. J. Cell Biol. 195, 369–376 10.1083/jcb.201102130 (doi:10.1083/jcb.201102130) 22024168 PMC3206335

[31] Yuzawa S Kamakura S Iwakiri Y Hayase J Sumimoto H 2011 Structural basis for interaction between the conserved cell polarity proteins Inscuteable and Leu–Gly–Asn repeat-enriched protein (LGN). Proc. Natl Acad. Sci. USA 108, 19 210–19 215 10.1073/pnas.1110951108 (doi:10.1073/pnas.1110951108) PMC3228485 22074847

[32] Knoblich JA Jan LY Jan YN 1999 Deletion analysis of the *Drosophila* Inscuteable protein reveals domains for cortical localization and asymmetric localization. Curr. Biol. 9, 155–158 10.1016/S0960-9822(99)80070-0 (doi:10.1016/S0960-9822(99)80070-0) 10021388

[33] Morin X Bellaiche Y 2011 Mitotic spindle orientation in asymmetric and symmetric cell divisions during animal development. Dev. Cell 21, 102–119 10.1016/j.devcel.2011.06.012 (doi:10.1016/j.devcel.2011.06.012) 21763612

[34] Kiyomitsu T Cheeseman IM 2012 Chromosome- and spindle-pole-derived signals generate an intrinsic code for spindle position and orientation. Nat. Cell Biol 14, 311–317 10.1038/ncb2440 (doi:10.1038/ncb2440) 22327364 PMC3290711

[35] Matsumura S Hamasaki M Yamamoto T Ebisuya M Sato M Nishida E Toyoshima F 2011 ABL1 regulates spindle orientation in adherent cells and mammalian skin. Nat. Commun. 3, 626 10.1038/ncomms1634 (doi:10.1038/ncomms1634) 22252550 PMC3324324

[36] Hao Y Du Q Chen X Zheng Z Balsbaugh JL Maitra S Shabanowitz J Hunt DF Macara IG 2010 Par3 controls epithelial spindle orientation by aPKC-mediated phosphorylation of apical Pins. Curr. Biol. 20, 1809–1818 10.1016/j.cub.2010.09.032 (doi:10.1016/j.cub.2010.09.032) 20933426 PMC2963683

[37] Siegrist SE Doe CQ 2005 Microtubule-induced Pins/Gαi cortical polarity in *Drosophila* neuroblasts. Cell 123, 1323–1335 10.1016/j.cell.2005.09.043 (doi:10.1016/j.cell.2005.09.043) 16377571

[38] Johnston CA Hirono K Prehoda KE Doe CQ 2009 Identification of an Aurora-A/PinsLINKER/Dlg spindle orientation pathway using induced cell polarity in S2 cells. Cell 138, 1150–1163 10.1016/j.cell.2009.07.041 (doi:10.1016/j.cell.2009.07.041) 19766567 PMC2789599

[39] Mauser JF Prehoda KE 2012 Inscuteable regulates the Pins-Mud spindle orientation pathway. PLoS ONE 7, e29611 10.1371/journal.pone.0029611 (doi:10.1371/journal.pone.0029611) 22253744 PMC3254608

[40] Speicher S Fischer A Knoblich J Carmena A 2008 The PDZ protein Canoe regulates the asymmetric division of *Drosophila* neuroblasts and muscle progenitors. Curr. Biol. 18, 831–837 10.1016/j.cub.2008.04.072 (doi:10.1016/j.cub.2008.04.072) 18499457

[41] Du Q Taylor L Compton DA Macara IG 2002 LGN blocks the ability of NuMA to bind and stabilize microtubules. A mechanism for mitotic spindle assembly regulation. Curr. Biol. 12, 1928–1933 10.1016/S0960-9822(02)01298-8 (doi:10.1016/S0960-9822(02)01298-8) 12445386

[42] Macara IG 2004 Parsing the polarity code. Nat. Rev. Mol. Cell Biol. 5, 220–231 10.1038/nrm1332 (doi:10.1038/nrm1332) 14991002

[43] Smith NR Prehoda KE 2011 Robust spindle alignment in *Drosophila* neuroblasts by ultrasensitive activation of pins. Mol. Cell 43, 540–549 10.1016/j.molcel.2011.06.030 (doi:10.1016/j.molcel.2011.06.030) 21855794 PMC3161515

[44] Pincet F 2007 Membrane recruitment of scaffold proteins drives specific signaling. PLoS ONE 2, e977 10.1371/journal.pone.0000977 (doi:10.1371/journal.pone.0000977) 17912354 PMC1991591

[45] Compton DA Luo C 1995 Mutation of the predicted p34cdc2 phosphorylation sites in NuMA impair the assembly of the mitotic spindle and block mitosis. J. Cell Sci. 108, 621–633 7769006 10.1242/jcs.108.2.621

[46] Galli M Munoz J Portegijs V Boxem M Grill SW Heck AJ van den Heuvel S 2011 aPKC phosphorylates NuMA-related LIN-5 to position the mitotic spindle during asymmetric division. Nat. Cell Biol. 13, 1132–1138 10.1038/ncb2315 (doi:10.1038/ncb2315) 21857670

[47] Tall GG Gilman AG 2005 Resistance to inhibitors of cholinesterase 8A catalyzes release of Gαi-GTP and nuclear mitotic apparatus protein (NuMA) from NuMA/LGN/Galphai-GDP complexes. Proc. Natl Acad. Sci. USA 102, 16 584–16 589 10.1073/pnas.0508306102 (doi:10.1073/pnas.0508306102) PMC1283842 16275912

[48] Woodard GE Huang NN Cho H Miki T Tall GG Kehrl JH 2011 Ric-8A and Gi α recruit LGN, NuMA, dynein to the cell cortex to help orient the mitotic spindle. Mol. Cell Biol. 30, 3519–3530 10.1128/MCB.00394-10 (doi:10.1128/MCB.00394-10) 20479129 PMC2897540

[49] Afshar K Willard FS Colombo K Johnston CA McCudden CR Siderovski DP Gonczy P 2004 RIC-8 is required for GPR-1/2-dependent Gα function during asymmetric division of *C. elegans* embryos. Cell 119, 219–230 10.1016/j.cell.2004.09.026 (doi:10.1016/j.cell.2004.09.026) 15479639

[50] Hampoelz B Knoblich JA 2004 Heterotrimeric G proteins: new tricks for an old dog. Cell 119, 453–456 10.1016/j.cell.2004.10.025 (doi:10.1016/j.cell.2004.10.025) 15537535

[51] David NB Martin CA Segalen M Rosenfeld F Schweisguth F Bellaiche Y 2005 *Drosophila* Ric-8 regulates Gαi cortical localization to promote Gαi-dependent planar orientation of the mitotic spindle during asymmetric cell division. Nat. Cell Biol. 7, 1083–1090 10.1038/ncb1319 (doi:10.1038/ncb1319) 16228010

[52] Thomas CJ Tall GG Adhikari A Sprang SR 2008 Ric-8A catalyzes guanine nucleotide exchange on Gα_i1_ bound to the GPR/GoLoco exchange inhibitor AGS3. J. Biol. Chem. 283, 23 150–23 160 10.1074/jbc.M802422200 (doi:10.1074/jbc.M802422200) PMC2516996 18541531

[53] Yoshiura S Ohta N Matsuzaki F 2012 Tre1 GPCR signaling orients stem cell divisions in the *Drosophila* central nervous system. Dev. Cell 22, 79–91 10.1016/j.devcel.2011.10.027 (doi:10.1016/j.devcel.2011.10.027) 22178499

